# Supplementary material for: Trans-2-Hexenal-Based Antifungal Packaging to Extend the Shelf Life of Strawberries
Source: Foods. 2021 Sep 13;10(9):2166. doi: 10.3390/foods10092166 (PMC8470944; doi:10.3390/foods10092166)
Supplement: Supplementary file 1 [file foods-10-02166-s001.zip › foods-1327316-supplementary.pdf]

Supplementary information: Preparation of active packaging systems

S1. Polyamide coating including HXAL

A polyamide coating containing a 10% of HXAL was applied on the surface of a PP film. PA was dissolved at 10% w/v concentration in 96% v/v ethanol under reflux at 80 ° C until polymer pellets were completely dissolved. After cooling the solution, 10% of HXAL (w/w of PA) was added. It was stirred for 15 min and a homogeneous solution was obtained. PP surface was cleaned with alcohol, corona treated with a BD-20AC laboratory corona treater (Electrotechnic Products, Chicago, IL) and coated. Coating was achieved by extending the PA solution on PP with a 100 µm coating rod (LinLab, Logroño, Spain) and drying using a hair dryer for 1 minute.

S2. Polyamide coating including HXAL previously encapsulated with cyclodextrins.

To reduce HXAL losses during coating development, the agent was previously complexed in β-cyclodextrins (β-CD). PA pellets were dissolved at 10% w/v in absolute ethanol under reflux at 80ºC. In parallel, βCD were dissolved in water at 10% w/v and HXAL was added at a 1:4 mass ratio (3:1 molar ratio) and keep under reflux at 60ºC. Both solutions were mixed in a ratio 4:1 (v/v) and stirred until cooled at room temperature. Corona treated PP film was coated with the above solution using 100 µm coating rod and drying using a hair dryer for 1 minute.

S3. Polyamide coating on paperboard including HXAL previously encapsulated with nanoclays.

HXAL was previously trapped in halloysite. Halloysite was dispersed in absolute ethanol at a concentration of 2%. Ultrasound was carried out in 3 cycles of 15 minutes. 1.0 % of HXAL was added and allowed to stand overnight. PA was then added to reach a concentration of 10% and stirred under reflux at 80 ° C until the solution was homogeneous. Coating was achieved by extending the PA solution on the unsized surface of paperboard with a 100 µm coating rod (LinLab, Logroño, Spain) and drying using a hair dryer for 1 minute

S4. HXAL injected in paperboard and coated with PA.

Since losses of trans-2-hexenal occurred during the coating drying process, a different methodology was attempted taking profit of the porous structure of paperboard. Pure HXAL was added at different volumes on paperboard. Folding carton was first coated with polyamide on the sized surface using a 10% PA solution in 96% ethanol and a 100 µm coating rod and dried with a hairdryer for 1 minute. Then, 250 µL of HXAL was deposited on 10 locations of the other paperboard surface (the unsized one). After adding these volumes, the surface was immediately coated with PA using the same process described above.

S5. HXAL injected in cellulosic pads and coated with PA.

A similar procedure was used to obtain active cellulosic pads. To produce this active device, first, an ethanol (96%) solution with 10% PA (w/v) was evenly spread onto the surface of one side of the pad using a 100 µm coating rod. This polymer was selected because PA provides a

high barrier to organic compounds in dry conditions, obtaining a good retention of HXAL, but a medium to low barrier when wet (presence of food), thus, facilitating its release [18]. The coating was dried in a homemade forced-air drying tunnel equipped with a 1000 W IR heat source for 2 min. Once dry, increasing volumes of the active compound—0, 100, and 250 µL—were injected into the cellulose pad equally distributed in 10 spots on the other surface and immediately covered with the same polymeric solution. Samples were identified as C (control), 100, and 250, respectively
